# Satisfaction with Life and Coping Strategies among Patients with Hidradenitis Suppurativa: A Cross-Sectional Study

**DOI:** 10.3390/jcm12082755

**Published:** 2023-04-07

**Authors:** Julia E. Rymaszewska, Piotr K. Krajewski, Łukasz Matusiak, Joanna Maj, Jacek C. Szepietowski

**Affiliations:** Department and Clinic of Dermatology, Allergology and Venerology, Wroclaw Medical University, 50-367 Wroclaw, Poland

**Keywords:** hidradenitis suppurativa, acne inversa, satisfaction with life, coping, coping mechanisms

## Abstract

Introduction: Hidradenitis suppurativa (HS) is a chronic recurrent inflammatory dermatosis with vast psychosocial burden. The objective of this study is to thoroughly analyze satisfaction with life (SWL) and coping strategies of HS patients in relation to the clinical and psychosocial factors. Methods: 114 HS patients (53.1% females; mean age 36.6 ± 13.1 years) were enrolled. Severity of the disease was measured using Hurley staging and International HS Score System (IHS4). Instruments utilized: Satisfaction with Life Scale (SWLS); Coping-Orientation to Problems-Experienced Inventory (Brief COPE); HS Quality of Life Scale (HiSQoL); Patient Health Questionnaire-9 (PHQ-9); Generalized Anxiety Disorder-7 (GAD-7); General Health Questionnaire (GHQ-28). Results: SWL was low in 31.6% of HS patients. No relation was found between SWL and Hurley staging and IHS4. SWL correlated with GHQ-28 (r = −0.579 *p* < 0.001), PHQ-9 (r = −0.603 *p* < 0.001), GAD-7 (r = −0.579 *p* < 0.001), and HiSQoL (r = −0.449 *p* < 0.001). Problem-focused coping strategies were most commonly used, followed by emotion-focused coping and avoiding coping strategies. Significant differences were found between the following coping strategies and SWL: self-distraction (*p* = 0.013), behavioral-disengagement (*p* = 0.001), denial (*p* = 0.003), venting (*p* = 0.019), and self-blame (*p* = 0.001). Conclusions: HS patients present low SWL which correlates with psychosocial burden. Reducing anxiety–depression comorbidity and encouraging optimal coping strategies may be of great importance in holistic approach to HS patients.

## 1. Introduction

Hidradenitis suppurativa (HS) is a disease of skin appendages and belongs to the spectrum of chronic inflammatory skin disorders. The condition presents itself in puberty with severely inflamed nodules, abscesses and draining tunnels, oozing skin lesions and intense scarring, which are localized in the armpits, under the breasts in women and in the anogenital area [1,2]. Aggravating, burdensome pain and itching as well as a malodourous discharge are the leading symptoms among patients with HS [3,4,5]. Up until now, the topic of the etiopathogenesis of this disease is being studied, although research on the main origin leans towards an autoinflammatory and genetic as well as a suspected hormonal background [6,7,8]. Additionally, HS patients usually present with other comorbidities such as, obesity and metabolic syndrome [9,10].

In the light of the above-described clinical manifestations, HS is likely to cause notable psychological distress [11,12,13,14,15]. Moreover, our recent study suggested that reasonable number of HS patients suffered from mental disorders, namely, depression and anxiety [16]. This in turn may lead to difficulties related to satisfaction with life (SWL), and utilization of different types of coping strategies [17]. However, the literature on the above-mentioned topics in HS patients is scarce [17]. 

Hence, the objective of the present study was to thoroughly analyze the satisfaction with life and coping strategies used in our cohort of HS patients.

## 2. Materials and Methods

### 2.1. Participants and Study Design 

Our study enrolled 114 consecutive patients suffering from HS. This cross-sectional study included 61 (53.3%) females and 53 (46.5%) males. The mean patients’ age was 36.6 ± 13.1 years (Table 1). Demographic and clinical data concerning the HS were collected. Moreover, the severity of HS was assessed by experienced dermatologists. Then, enrolled patients filled out the below given set of questionnaires in validated Polish language versions.

This study received approval from the Wroclaw Medical University Ethics Committee (KB-901/2022). The data were collected from two different regions of Poland (southwest and southeast Poland) between September 2020 and September 2021. 

### 2.2. Assessments 

#### 2.2.1. HS Severity 

Hurley staging [18] and International Hidradenitis Suppurativa Severity Score System (IHS4) [19] were utilized in assessing staging and clinical severity of HS. Hurley staging is a widely used grading system to characterize the extend of HS lesions. It categorizes HS patients into 3 groups based on the presence and extend of lesions, scaring and sinus tracts. Hurley stage I presents as inflammatory nodule or abscess formation, single or multiple without scaring and sinus tracts. Hurley stage II groups with recurrent abscesses or nodules with sinus tract formation and scaring. Typically, multiple separated lesions are present. Hurley stage III presents as diffuse involvement with multiple interconnected sinus tracts, scaring and abscesses covering entire area [18]. IHS4 is a validated tool to assess clinical severity of HS calculated with the following formula: IHS4 (points) = (number of nodules multiplied by 1) + (number of abscesses multiplied by 2) + (number of draining tunnels multiplied by 4) [19]. The following cut-off points of IHS4 were used to classify the patients into different severity groups: up to 3 points—mild HS; 4–10 points—moderate HS; and above 10 points—severe HS [18,19]. 

#### 2.2.2. Satisfaction with Life

Satisfaction with life was assessed using the Satisfaction with life Scale (SWLS) [20]. SWLS is a 5-item scale, where a patient estimates to what extent each of them relates to his/her life so far, rated on a 7-point scale from 1 point—“I completely disagree” to 7 points—“I completely agree”. The overall score is the sum of all grades. The range of results ranges from 5 to 35 points, the higher the score, the greater the sense of SWL. In order to determine the sense of SWL, the values were converted to a sten scale. In the interpretation of the results, the values in the range from 1 to 4 points are presented as low, the results in the range of 5 to 6 points are presented as average, and those in the range from 7 to 10 points are presented as high SWL [20].

#### 2.2.3. Coping Strategies

A shortened version of the Coping Orientation to Problems Experienced Inventory, Brief COPE was employed [21]. It is a tool for measuring coping strategies, assessing typical ways of reacting and feeling in situations of experiencing severe stress. It involves 28 statements that are included in 14 coping strategies (2 statements in each strategy). The following strategies are analyzed: active coping (items 2, 7), planning (items 14, 25), positive reframing (items 12, 17), acceptance (items 20, 24), sense of humor (items 18, 28), turning to religion (items 22, 27), seeking emotional support (items 5, 15), seeking informational support (items 10, 23), self-distraction (items 1, 19), denial (items 3, 8), venting (items 9, 21), substance use (items 4, 11), behavioral disengagement (items 6, 16) and self-blame (items 13, 26). For each statement, the respondent marks 1 out of 4 possible answers, which are scored: “I almost never do this” (0 points), “I rarely do this” (1 point), “I often do this” (2 points) and “ I almost always do that “(3 points). Each of the 14 coping strategies is assessed separately by adding together the points for the answers to the two statements that make it up and dividing the sum by 2. Out of 14 strategies, 3 main domains of strategies were distinguished as subscales: problem-focused coping (active coping, seeking informational support, positive reframing and planning), emotion-focused coping (seeking emotional support, venting, sense of humor, acceptance, turning to religion and self-blame) and avoidant coping (self-distraction, denial, substance use and behavioral disengagement). The higher the score, the more often the test person uses a given strategy [22].

#### 2.2.4. Psychopathological Symptoms

The General Health Questionnaire (GHQ-28) [23] is a 28-item scale utilized for screening of minor psychiatric and non-psychotic disorders. It is divided into 4 subscales: somatic symptoms (items 1–7); anxiety/insomnia (items 8–14); social dysfunction (items 15–21); and severe depression (items 22–28). The GHQ-28 can be scored from 0 to 3 points for each response (total possible score on the ranging from 0 to 84 points) [23].

Mental status of the participants in the last 14 days was additionally assessed with Patient Health Questionnaire-9 (PHQ-9) [24] and the Generalized Anxiety Disorder-7 (GAD-7) [25]. Each item of both scales can be scored from 0 to 3 points (0 points—not at all, 1 point—several days, 2 points—more than half days, 3 points—nearly every day). The PHQ-9 scale includes 9 items about feeling sad, depressed, or hopeless, sleep disturbance, lack of energy, appetite changes, problems with focusing on certain tasks and thoughts about hurting oneself or death. The PHQ-9 total score ranges between 0 and 27 points. The GAD-7 has 7 questions to evaluate the sense of anxiety, tension, nervousness, the ability to control these feelings, the ease with which they appear and problems with relaxing. The GAD-7 total score ranges between 0 and 21 points. The higher the scores for both scales the higher risk for the development of depression (PHQ-9) and anxiety (GAD-7) [24,25].

#### 2.2.5. Quality of Life

HS-specific quality of life was measured with Hidradenitis Suppurativa Quality of Life Scale, HiSQoL [26]. HISQoL is 17-item scale evaluating HS patients’ quality of life within the last 7 days. It contains a 5-point item tool which consolidates responses such as “extremely”, “very much”, “moderately”, “slightly” and “not at all” with answers scored 4, 3, 2, 1 and 0 points, respectively [17]. Additionally, subsidiary items such as “unable to do, due to my HS” (score 4 points) and/or “I do not normally do this, HS did not influence” (score 0 points) were given. Moreover, this questionnaire was divided into three subscales: activities–adaptations, psychosocial and symptoms [27]. The higher the score of the questionnaire, the greater the decreased quality of life of HS patients.

### 2.3. Statistical Analysis

The statistical analysis of the obtained results was performed using IBM SPSS Statistics v. 26 (SPSS INC., Chicago, IL, USA) software. All the data were assessed for normal or non-normal distribution. The minimum, maximum, mean, standard deviation, median and quartiles were calculated. Analyzed quantitative variables were evaluated depending on the normality using the T-student or Mann–Whitney U test. The correlations between variables were assessed with Spearman or Pearson correlations. The Chi2 test was used for the comparison of qualitative data. Differences in coping strategies between different HS severity stages (assessed with Hurley and IHS4), as well as for the different levels of satisfaction with life were assessed using Kruskal-Wallis’s 1-way analysis of variance on ranks with the post hoc analysis according to the Bonferroni correction. A 2-sided *p* value ≤ 0.05 was considered to be statistically significant.

## 3. Results

### 3.1. Clinical HS Severity

According to Hurley staging the majority of our patients, 75 subjects (65.8%) presented with Hurley stage II, 25 patients (21.9%) patients were diagnosed with Hurley stage I and the remaining 14 (12.3%) with Hurley stage III. The mean clinical severity of the disease assessed with IHS4 among our study group was 14.6 ± 17.0 points. In relation to cut-off points of IHS4, 26 patients (22.8%) suffered from mild HS, 42 (36.8%) from moderate HS and 46 subjects (40.4%) had severe disease. Mean duration of HS was 9.76 ± 8.20 years (Table 1).

### 3.2. Satisfaction with Life

Among our group of HS patients, 36 subjects (31.6%) reported low SWL. Average SWL was found in 48 patients (42.1%) and the remaining ones—30 patients (26.3%)—showed high SWL. 

Results presented in Table 2 show that SWL of both female and male subjects with HS were practically the same. No statistically significant difference was found in SWL between different HS severity groups (both Hurley and IHS4). Additionally, there was no correlation between SWLS and IHS4 scores. Moreover, SWLS did not correlate with patients’ age, number of hospitalizations due to HS and the duration of the disease.

**Figure 1:**
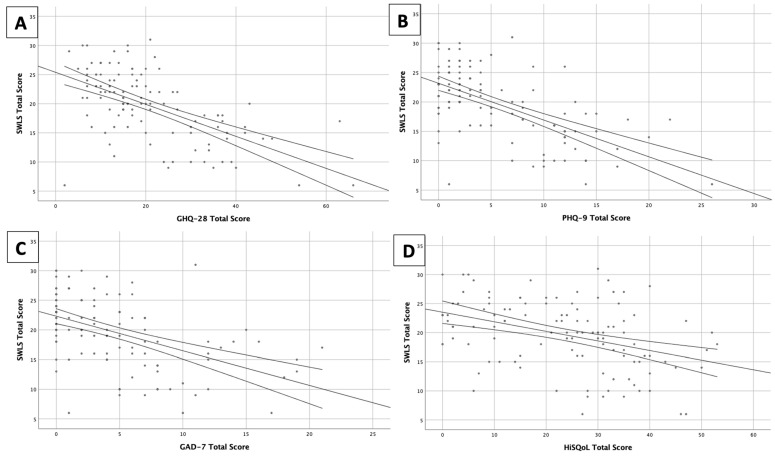
Correlations between satisfaction with life and (**A**) GHQ-28, (**B**) PHQ-9, (**C**) GAD-7 and (**D**) HiSQoL. Each dot represents each patient’s questionnaire score.

There was a moderate negative correlation (r = −0.579 *p* < 0.001) between SWLS and psychopathological symptoms, measured by the GHQ-28. A strong negative correlation (r = −0.603 *p* < 0.001) between SWLS and depressive symptoms (PHQ-9) among our HS patients was also found. Moreover, a moderate negative correlation (r = −0.579 *p* < 0.001) was demonstrated between SWLS and anxiety symptoms (GAD-7) in HS subjects. Additionally, SWL significantly moderately negatively correlated (r = −0.449 *p* < 0.001) with HS-specific quality of life measured by HiSQoL (Figure 1). 

Taking into consideration different domains of HiSQoL, there was a moderate significant negative correlation (r = −0.478, *p* < 0.001) between SWLS and psychosocial part of HiSQoL questionnaire. SWL of our HS subjects also correlated negatively weakly with the remaining domains of HiSQoL: activities–adaptation and symptoms (r = −0.366 *p* < 0.001 and r = −0.331, *p* = 0.011, respectively). The same above correlations were observed separately for female and male HS patients (Table 3). 

### 3.3. Coping Strategies

**Figure 2:**
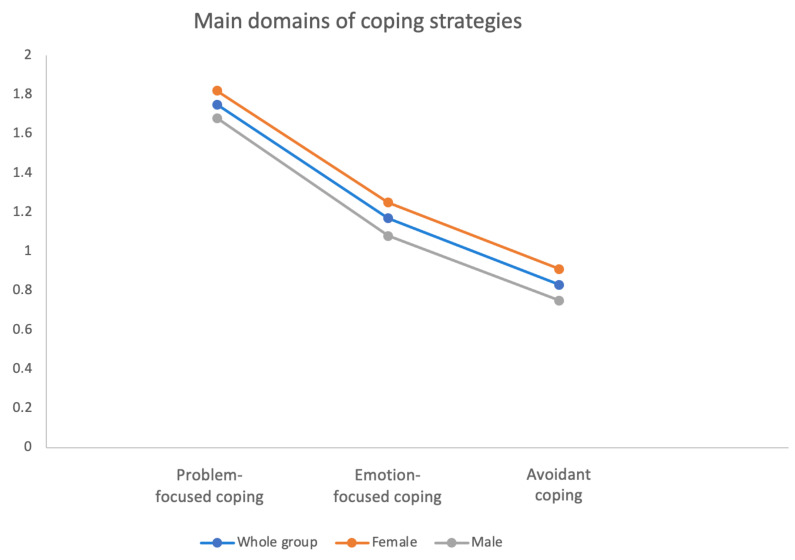
Frequency of utilizing of main domains of coping strategies.

**Figure 3:**
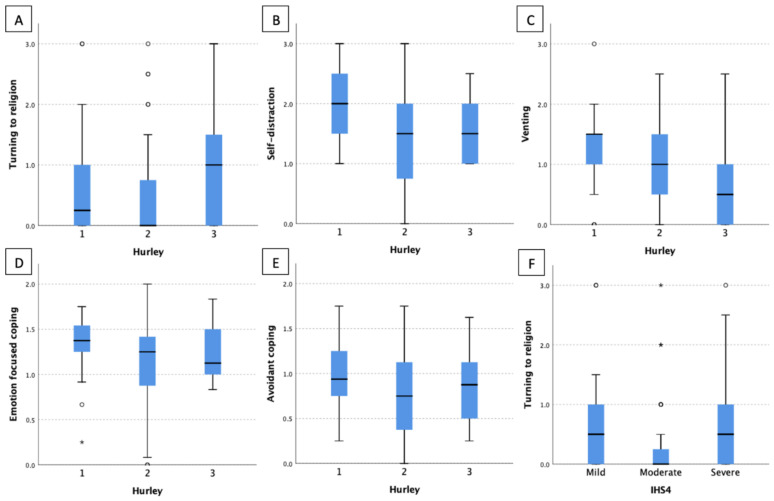
Coping strategies in different HS severity. (**A**) turning to religion/Hurley; (**B**) self-distraction/Hurley; (**C**) venting/Hurley; (**D**) emotion focused coping/Hurley; (**E**) avoidant coping/Hurley; (**F**) turning to religion/IHS4. Each dot represents each patient’s questionnaire score, while asterisks represent outlier data.

Among HS patients studied, patients’ problem-focused coping strategies were most commonly used, followed by emotion-focused coping and avoiding coping strategies (Table 4). There was no difference in the use of main domains of coping strategies between females and males (Figure 2). Concerning particular coping strategies active coping, planning, acceptance, seeking emotional support and seeking informational support were most frequently used by the entire group of patients (Table 4). After stratifying the results according to gender, seeking informational support (*p* = 0.039) and self-distraction (*p* = 0.028) were strategies utilized more frequently by females than males (Table 4). There were significant differences in utilizing emotion-focused coping and avoidant coping mechanisms among patients with different Hurley staging groups (*p* = 0.027 and *p* = 0.034), respectively. Patients with Hurley I used both above-mentioned strategies more often than the Hurley II patients (*p* = 0.022 and *p* = 0.028, respectively) and Hurley III subjects (differences not significant) (Figure 3). Moreover, additional differences were disclosed in particular coping strategies between patients with different HS severities. Turning to religion tended to be most frequently used in patients with the most severe disease (*p* = 0.036 for Hurley staging and *p* = 0.034 for IHS4). Self-distraction and venting appeared to be more commonly implemented among patients with Hurley I disease (*p* = 0.026 and *p* = 0.037, respectively). No other significant difference in types of coping mechanisms and disease severities were found (Figure 3). 

### 3.4. Relationship between Satisfaction with Life and Coping Strategies

**Figure 4:**
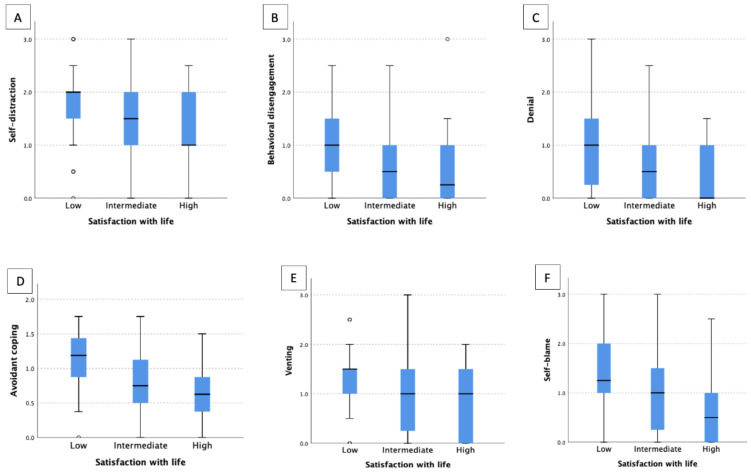
Coping strategies in HS patients with various satisfaction with life. (**A**) self-distraction/SWL; (**B**) behavioral disengagement/SWL; (**C**) denial/SWL; (**D**) avoidant coping/SWL; (**E**) venting/SWLl (**F**) self-blame/SWL. Each dot represents each patient’s questionnaire score.

We identified the significant differences between SWL and the following coping strategies: self-distraction (*p* = 0.013), behavioral disengagement (*p* = 0.001), denial (*p* = 0.003), venting (*p* = 0.019) and self-blame (*p* = 0.001) (Figure 4). Thus, patients with high SWL tended to utilize self-distraction less frequently (*p* = 0.012) than patients with low SWL. Subjects with intermediate and high SWL used behavioral disengagement more sporadically than patients with low SWL (*p* = 0.004 and *p* = 0.002, respectively). Moreover, HS patients with low SWL utilized denial more often (*p* = 0.002) in comparison to subjects with high SWL. Consequently, HS subjects with low SWL made use of the whole coping domain, i.e., avoidant coping, more often than those with intermediate and high SWL (*p* = 0.002 and *p* < 0.001, respectively). Furthermore, venting was used more often (*p* = 0.036) amid participants with low SWL than with high SWL. Additionally, HS patients with low SWL tended to employ self-blame more often than subjects with intermediate and high SWL (*p* = 0.043 and *p* = 0.001, respectively). 

## 4. Discussion

In our study, we focused on assessing factors like satisfaction with life and coping strategies in a group of HS patients in southwest and southeast Poland. The newest research shows that in Europe, HS prevalence ranges from 0.001 to 1.4% [28]. Perhaps these big discrepancies are related to a growing awareness of the disease. An example can be an Italian study by Bettoli et al. [29]. They presented an investigation of potential differences between two successive Italian HS patient registries 2009–2013 vs. 2015–2019 and came to the conclusion that the illness frequency estimations were growing from 5:10,000 to 4:100 people, respectively [29].

Despite the importance of SWL and coping mechanisms, these elements seem to have attracted insufficient attention in the scientific literature [17]. To handle the emotional pressures experienced by individuals with chronic illnesses, patients must learn to regulate their emotions, which involves experiencing, processing and modifying their emotional reactions. The resources required for daily self-care management of chronic disease are depleted by overwhelming emotional demands, which has a negative impact on health outcomes [30]. The diagnosis of HS is known to have a major negative impact on mental health, leading to long-term psychological suffering characterized, among other psychopathological variables, by depressive or anxiety symptoms [14,31]. To our knowledge, this is the first study to identify how demographic and clinical characteristics relate to the chronic disease, as well as how coping mechanisms relate to the overall life satisfaction of patients with HS. 

A paper conducted by Matusiak et al. [13], utilizing a different tool which included satisfaction of life (Quality of Life Enjoyment and Satisfaction Questionnaire Short Form O-LES-Q-SF) among HS patients, demonstrated no significant correlation between questionnaire-related scores and patient’s gender and duration of the HS disease, similar to our investigation. Their results showed that the patients with the most severe HS (Hurley III) had significantly lower O-LES-Q-SF scores [13]. They could be considered different from our findings; however, Matusiak et al. [13] evaluated a complex Quality of Life paradigm, so it is difficult to compare these results directly. Additionally, it is noteworthy that Matusiak et al. [13] results may differ to ours since their study with a lower sample size consisted of significantly more HS patients with Hurley stage III (22.2% in comparison to ours 8.8%). A similar study on SWL in chronic dermatoses was conducted by Soliman et al. [32] on psoriatic patients. Their results (based on the same Satisfaction with Life Scale, SWLS) showed a quite similar average SWL in psoriatic patients in comparison to our HS group (mean: 21.2 points and 19.8 points, respectively). Moreover, the results of our study are in agreement with Soliman et al.’s [32] findings concerning lack of influence of gender, severity and duration of the disease on SWL [32]. 

The high rate (appr. 1/3 of respondents) of low SWL among our HS patients is worrying. Investigation of the possible reasons for this outcome should be crucial. Our research shows that factors other than sociodemographic factors related to the disease and its severity are important for the determination of the level of SWL. Additionally, taking into account the structure of the SWLS, which contains general questions regarding the assessment of SWL, skin disease does not seem to be the main or the most significant factor influencing the SWL. Many causes can be possible, including psychological ones, personality traits and social relations in private and work life, which were not analyzed in our study. In our research, we took into account a very important factor, one that is directly influencing the assessment of SWL, which is the mental state of the respondents at the time of filling out the questionnaires. Our study revealed that lower SWL was directly affected by the higher severity of mental symptoms such as anxiety and depression among our respondents. 

Kowalewska et al. [33] conducted a study on psoriatic patients and assessed relationships between the acceptance of illness, quality of life and SWL [33]. The respondents evaluated their SWL as low (37%), where our results yielded 31.6% of low SWL in the group of our HS patients. Additionally, Kowalewska et al. [33] showed that levels of SWL in both genders slightly differ. In our paper, we found no significant differences among SWL between patients of both genders. In comparison to Kowalewska et al. [33], where only 18.13% of females had moderate SWL, our group presented 39.4% patients with intermediate SWL. Further, high SWL in our male group was only 26.4%; in Kowalewska et al.’s [33] study, it was 48.48%. 

When comparing the level of SWL in two chronic diseases assessed based on our study and that of Kowalewska et al. [33], we can posit that the differences in SWL outcomes can be justified by the fact that the age of both measured groups was slightly different, as well as the different number of patients analyzed (psoriasis N = 366 and HS N = 114) [33].

A high percentage (62%) of patients with low SWL suffering from systemic lupus erythematosus was found by Kulczycka et al. [34] This result is almost twice as high as in our study (31.6%). They utilized the same SWLS [34]. In both studies, the number of participants and their age groups were similar. Kulczycka et al. [34] also found a lack of significant correlation between the severity of systemic lupus erythematosus symptoms and SWL. This leads us to hypothesize that the type of a chronic disease, especially the ones which manifest themselves not only dermatologically but also systemically, influences the level of life satisfaction of the patients.

The findings of our study provide a significant contribution to our understanding of patient SWL and coping mechanisms. The outcomes concerning the coping strategies of the population are supported by other research on other chronic diseases, not only those of a dermatological nature. Coping strategies are a relatively stable feature for an individual, not very susceptible to fluctuations and changes over time. Many individuals rely not only on one coping strategy, but on several different ones over their lifetime [35].

Among our group of patients, the most often utilized coping strategies were active coping, planning and acceptance. The findings of this study are in accordance with the paper by Richards et al. [36]. They analyzed coping strategies in a different disease of a dermatological nature—polymorphic light eruption. Their results, based also on the Brief-COPE questionnaire, demonstrated that the most often used adaptive strategies were: acceptance (76%), active coping (67%) and planning (60%) [36]. 

Our current study concerning coping strategies may find further explanation in a previous study by Finzi et al. [22]. They aimed to define the prevalence of psychopathological distress and coping mechanisms among patients with psoriasis. They utilized the same Brief-COPE scale and a shortened version of GHQ-28 scale—GHQ-12. Their results showed that the highest scores were obtained in two main strategies: planning and active coping. Additionally, in a cohort analyzed by Finzi et al. [22], men had notably higher scores than women in strategies such as self-distraction, venting, religion, use of emotional support and denial [22]. It is noteworthy that our study on a different chronic and debilitating dermatosis—namely, HS—yielded similar results concerning the general group of HS patients. However, in our paper, after stratifying the results according to gender, we established that seeking informational support and self-distraction were strategies utilized more frequently by women than men. 

Researchers have also evaluated coping mechanisms with the help of the Brief-COPE scale among patients with inflammatory bowel disease. The pattern revealed that emotion-focused coping was the predominantly utilized mechanism [37]. This is partially in agreement with our clinical sample. We established that certain coping strategies, belonging to two different strategies (problem-focused and emotional-focused coping), were most frequently used by the entire group of our HS patients. Problem-focused coping includes strategies that aim to alter or remove a stressor. However, emotion-focused coping incorporates dealing with the stressor while utilizing one’s emotional responses [38]. Therefore, we hypothesize that choosing certain coping strategies by HS patients can be influenced by current circumstances and common HS symptoms, such as odor, sourness and the visibility of lesions. 

Our current study identified significant differences between SWL and certain coping strategies. Self-distraction was therefore used by patients with high SWL less frequently in comparison to those with low SWL. In contrast to patients with low SWL, subjects with intermediate and high SWL sporadically employed the behavioral disengagement strategy in comparison to patients with low SWL. Denial was also used more frequently by HS patients with low SWL compared to participants with high SWL. HS individuals with low SWL used the avoidant coping domain more frequently than those with moderate and high SWL. A study by Ziarko et al. [39] measured the role of coping and life satisfaction among patients with a different chronic disease such as rheumatoid arthritis. They established that strategies such as turning to religion, seeking emotional support and denial were the primary determinants of the level of their life satisfaction. Yet another relevant outcome of our study is that the patients who utilize denial as a coping mechanism were less satisfied with their lives. Ziarko et al. [39], however, presented results stating that the patients adopting active coping strategies and self-distraction are more satisfied with their lives [39]. 

Among patients suffering from multiple sclerosis, a chronic and debilitating neurological disease, acceptance, planning and positive reframing strategies were shown to moderate the distress caused by the disease. It was suspected by the authors that a certain way of perceiving coping with the disease by patients with multiple sclerosis might be due to the fact that the main additional burden was the COVID-19 pandemic [40]. 

Furthermore, interesting findings regarding SWL and coping strategies were established by Blaževi et al. [41]. In their paper, patients suffering from chronic urticaria were less satisfied with their lives in comparison to ones with acute urticaria. Additionally, patients with acute urticaria used turning to religion, seeking emotional support and denial strategies to a greater degree compared to patients with chronic urticaria. Numerous research has shown that the purpose of emotion-focused coping is to lessen or ease the emotional arousal brought on by a stressful circumstance, in this case—acute urticaria [42]. We hypothesize that among these patients, choosing more emotional strategies indicates high emotionality, which, in turn, can lead to an acute urticaria appearing in a more stressful situation. In chronic urticaria, patients might choose emotional strategies less often, which suggests that in chronic urticaria, the emotional response to stress may be less important in the chronic course of illness. 

We are aware of the limitations of our study. HS is rare condition, and we ran the study only in two regions of our country. It will be worth confirming our findings in the multicenter study involving more HS patients in the future. Moreover, the data were collected only by utilizing self-reported questionnaires. The screening of the mental status was not confirmed with detailed psychiatric examination. Moreover, the data were collected only in two centers. We are unable to determine the exact reason for the lower SWL of the surveyed patients. We have not analyzed SWL in relation to location of the HS lesions, including the anogenital region. It is probably that factors other than those related to the disease might have had an influence. 

## 5. Conclusions

In conclusion, we clearly documented low SWL in the reasonable number of HS patients, which significantly correlated with numerous psychosocial parameters. Moreover, we found that different coping strategies were utilized by different HS subgroups. All measured modalities are patient-centered and assess both mental state, quality of life and coping with the disease. Taking into consideration the fact that the utilized tools are self-assessment scales, medicine is moving towards greater personalization and a holistic patient approach. Today patients’ feelings and afflictions play a bigger role in care than raw test results. We think that further research in this area is required, particularly with a group of individuals who have more severe HS symptoms; it will provide a more accurate evaluation of how the condition affects both coping and overall SWL. Consequently, it is of great importance to establish interventions that can improve life with HS. Finding the psychological elements that affect coping appears to be essential. Reducing anxiety–depression comorbidities and encouraging optimum coping may be the main goals for these patients’ improvements in the absence of a cure. However, long-term studies to assess coping efficacy and SWL in this patient population should be just as important.

## Figures and Tables

**Table 1:** Demographic and clinical characteristics of HS patients.

Characteristics	Whole Group (*n* = 114)	Females (*n* = 61)	Males (*n* = 53)	*p*
Sex, number of participants (%)		NA	NA	NA
Males	53 (46.5)			
Females	61 (53.5)			
Age Mean ± SD (years)	36.56 ± 13.10	37.47 ± 13.63	39.92 ± 12.5	NS
Duration of the disease Mean ± SD (years)	9.76 ± 8.20	10.9 ± 7.99	8.57 ± 8.38	0.028
Number of hospitalizations, Mean ± SD (years)	1.7 ± 2.63	1.86 ± 3.23	1.51 ± 1.66	NS
Hurley stages, *n* (%)				
I	28 (24.6)	17 (27.9)	11 (20.8)	NS
II	76 (66.7)	41 (67.2)	35 (66.0)	NS
III	10 (8.8)	3 (4.9)	7 (13.2)	NS
IHS4 severity stage, *n* (%)				
Mild	26 (22.8)	15 (24.6)	11 (20.8)	NS
Moderate	40 (35.1)	21 (34.4)	19 (35.8)	NS
Severe	48 (42.1)	25 (41.0)	23 (43.4)	NS

*n*—number of patients; SD—standard deviations; NA—not applicable; NS—not significant; IHS4—International Hidradenitis Suppurativa Score System.

**Table 2:** Satisfaction with life among patients with hidradenitis suppurativa.

SWLS	Whole Group (*n* = 114)	Females (*n* = 61)	Males (*n* = 53)	*p*
Total score, mean ± SD	19.8 ± 5.7	19.53 ± 5.94	19.7 ± 5.7	NS
SWL, *n* (%)				
Low Intermediate High	36 (31.6) 48 (42.1) 30 (26.3)	21 (34.4) 24 (39.4) 16 (26.2)	15 (28.3) 24 (45.3) 14 (26.4)	NS

*n*—number of patients; SD—standard deviations; NA—not applicable; NS—not significant; SWL—satisfaction with life; SWLS—Satisfaction with Life Scale.

**Table 3:** Correlations of satisfaction with life with different psychosocial aspects.

SWLS Total Score	GHQ-28	PHQ-9	GAD-7	HiSQoL Total Score	HiSQoL Activities–Adaptations	HiSQoL Symptoms	HiSQoL Psychosocial
Whole group (*n* = 114)	r = −0.579 *p* < 0.001	r = −0.603 *p* < 0.001	r = −0.579 *p* < 0.001	r = −0.449 *p* < 0.001	r = −0.366 *p* < 0.001	r = −0.331 *p* = 0.011	r = −0.478 *p* < 0.001
Females (*n* = 61)	r = −0.533 *p* < 0.001	r = −0.699 *p* < 0.001	r = −0.624 *p* < 0.001	r = −0.455 *p* < 0.001	r = −0.364 *p* = 0.005	r = −0.455 *p* < 0.001	r = −0.458 *p* < 0.001
Males (*n* = 53)	r = −0.636 *p* < 0.001	r = −0.507 *p* < 0.001	r = −0.549 *p* < 0.001	r = −0.483 *p* < 0.001	r = −0.406 *p* = 0.003	r = −0.305 *p* = 0.026	r = −0.497 *p* < 0.001

SWLS—Satisfaction with Life Scale; GHQ-28—General Health Questionnaire 28; PHQ-9—Patient Health Questionnaire 9; GAD-7—Generalized Anxiety Disorder 7; HiSQoL—Hidradenitis Suppurativa Quality of Life; *n*—number of patients.

**Table 4:** Differences in coping strategies among patients with hidradenitis suppurativa.

Coping Strategies Mean ± SD	Whole Group (*n* = 114)	Females (*n* = 61)	Males (*n* = 53)	*p*
Problem-focused coping Active coping Planning Positive reframing Seeking informational support	1.75 ± 0.65 1.96 ± 0.85 1.93 ± 0.88 1.52 ± 0.76 1.61 ± 0.84	1.82 ± 0.63 1.98 ± 0.85 1.95 ± 0.87 1.58 ± 0.74 1.76 ± 0.83	1.68 ± 0.68 1.92 ± 0.87 1.92 ± 0.9 1.44 ± 0.79 1.6 ± 0.87	NS NS NS NS 0.039
Emotion-focused coping Acceptance Sense of humor Turning to religion Seeking emotional support Venting Self-blame	1.17 ± 0.65 1.84 ± 0.8 0.89 ± 0.63 0.54 ± 0.79 1.76 ± 0.8 1.01 ± 0.75 0.98 ± 0.82	1.25 ± 0.38 1.97 ± 0.73 0.87 ± 0.64 0.59 ± 0.85 1.89 ± 0.72 1.12 ± 0.71 1.07 ± 0.92	1.08 ± 0.47 1.7 ± 0.86 0.91 ± 0.62 0.47 ± 0.7 1.61 ± 0.87 0.88 ± 0.77 0.89 ± 0.68	NS NS NS NS NS NS NS
Avoidant coping Self-distraction Denial Substance use Behavioral disengagement	0.83 ± 0.46 1.53 ± 0.85 0.66 ± 0.7 0.46 ± 0.74 0.68 ± 0.7	0.91 ± 0.45 1.71 ± 0.8 0.68 ± 0.8 0.52 ± 0.83 0.73 ± 0.71	0.75 ± 0.47 1.33 ± 0.88 0.64 ± 0.57 0.39 ± 0.63 0.63 ± 0.7	NS 0.028 NS NS NS

SD—standard deviation; *n*—number of patients; NS—not significant.

## Data Availability

Data supporting the reported results can be obtained on request, e-mail: julia.rymaszewska@umw.edu.pl.
